# A Comprehensive Analysis of the CaMK2A Gene and Susceptibility to Alzheimer’s Disease in the Han Chinese Population

**DOI:** 10.3389/fnagi.2019.00084

**Published:** 2019-04-11

**Authors:** Xinyu Fang, Wei Tang, Fuyin Yang, Weihong Lu, Jun Cai, Jianliang Ni, Jiangtao Zhang, Wenxin Tang, Tao Li, Deng-Feng Zhang, Chen Zhang

**Affiliations:** ^1^Shanghai Mental Health Center, Shanghai Jiao Tong University School of Medicine, Shanghai, China; ^2^Wenzhou Kangning Hospital, Wenzhou Medical University, Wenzhou, China; ^3^Tongde Hospital of Zhejiang Province, Hangzhou, China; ^4^Hangzhou Seventh People’s Hospital, Hangzhou, China; ^5^Huaxi Brain Research Centre, West China Hospital, Sichuan University, Sichuan, China; ^6^Key Laboratory of Animal Models and Human Disease Mechanisms, Kunming Institute of Zoology, Chinese Academy of Sciences, Yunnan, China

**Keywords:** Alzheimer’s disease, CaMK2A, gene, haplotype, eQTL, China

## Abstract

There is ample evidence suggesting that calcium/calmodulin-dependent protein kinase II alpha (CaMK2A) may play an important role in the pathophysiology of Alzheimer’s disease (AD). This genetic study aimed to investigate whether *CaMK2A* confers susceptibility to the development of AD in the Han Chinese population. A total of seven single nucleotide polymorphisms (SNPs) within *CaMK2A* were screened in two independent cohorts from southwestern China (333 AD patients and 334 controls) and eastern China (382 AD patients and 426 controls) to discern the potential association between this gene and AD. In addition, a cross-platform normalized expression resource was used to investigate whether *CaMK2A* is differentially expressed in the brain between individuals with AD and the controls. In addition, expression quantitative trait loci (eQTL) analysis was used to explore the differences in *CaMK2A* expression in the brain among different genotypes. The cross-platform normalized data showed significant differences in *CaMK2A* expression in the hippocampus, entorhinal cortex and temporal cortex between the AD patients and the control subjects (|log FC| > 0.1, *P* < 0.05); however, only the differences in the hippocampus and temporal cortex remained after the multiple comparisons correction [false discovery rate (FDR)-corrected, *P* < 0.05]. The frequency of the rs4958445 genotype was significantly different between the AD subjects and the controls from southwestern China (*P* = 0.013, *P* = 0.034 after FDR correction). When the two samples were combined, rs4958445 still showed a significant association with AD (*P* = 0.044). Haplotype analysis indicated that the T-A-C-A-T-C-C and T-G-C-A-T-C-C haplotypes in the southwestern cohort and the T-G-C-G-C-T-C haplotype in the eastern cohort, consisting of rs10051644, rs6869634, rs3797617, rs3756577, rs4958445, rs10515639 and rs6881743, showed a significant association with AD (*P* = 0.037, *P* = 0.026 and *P* = 0.045, respectively). Furthermore, the brain eQTL analysis revealed a significant association between the rs4958445 polymorphism and *CaMK2A* expression in the inferior olivary nucleus (*P* = 0.029). Our results suggest an important role for *CaMK2A* in the pathophysiology of AD in the Han Chinese population, especially the southwestern population.

## Introduction

Alzheimer’s disease (AD), which is the most common neurodegenerative disorder, is primarily characterized by progressive cognitive impairment in the elderly (Zhang D. F. et al., 2016; Xing et al., 2017). Epidemiological studies have demonstrated that AD will become more prevalent by the middle of this century and therefore represents a major global public health problem (Frozza et al., 2018). Although the underlying cause of AD is unclear in most cases, numerous genetic alterations have been identified as being associated with the risk of AD (Wang et al., 2016a,b; Zhang D. F. et al., 2016; Li G. D. et al., 2017; Tang et al., 2017), and signaling pathways involved in memory loss in AD are also under intense investigation (Vázquez-Higuera et al., 2011; Berridge, 2016).

It has been well established that altered synaptic Ca^2+^ signaling may be involved in the pathogenesis of AD (Jang and Chung, 2016). Calcium/calmodulin-dependent protein kinase II (CaMK2) is a multifunctional protein kinase that is highly expressed in the central nervous system (CNS) and is activated by the binding of Ca^2+^/calmodulin (Lisman et al., 2012). Early literature reported that CaMK2 plays a crucial role in gene expression, memory processing, learning and neuroplasticity in the CNS (Irvine et al., 2006; Lisman et al., 2012; Wang D. et al., 2018). The role of CaMK2A is especially important, as this subunit is the most abundant subunit of CaMK2 in the brain (Hanson and Schulman, 1992). There is accumulating evidence indicating that CaMK2A is required for long-term potential (LTP), which is the long-lasting form of synaptic plasticity and is required for memory formation (Irvine et al., 2006). Preclinical studies demonstrated that CaMK2A knock-in mutant mice have impaired memory and LTP (Giese et al., 1998; Yamagata et al., 2009). Moreover, postmortem analyses also confirmed that CaMK2A is dysregulated in the hippocampus in AD, and CaMK2A-expressing neurons are selectively lost at synaptic locations in the hippocampus of AD patients (Wang et al., 2005; Reese et al., 2011). These results suggest that aberrant synaptic CaMK2A activity may play an important role in the pathogenesis of AD.

The gene encoding CaMK2A is located at human chromosome 5q32. A recent study reported that the *CaMK2A* rs3756577 and rs3822606 polymorphisms confer susceptibility to AD in the Spanish population (Bufill et al., 2015), whereas earlier literature did not support the association of *CaMK2A* with AD in this population (Vázquez-Higuera et al., 2011). To the best of our knowledge, no genetic study to date has addressed the association of *CaMK2A* with AD in the Chinese Han population.

In the present study, we attempted to explore the potential association between common variants of* CaMK2A* and the genetic risk of AD in two independent Han Chinese samples from eastern and southwestern China. First, a public database was used to determine whether *CaMK2A* is differentially expressed in the brain between AD patients and healthy controls. Second, we completely genotyped seven* CaMK2A* single nucleotide polymorphisms (SNPs) in our samples. Third, brain expression quantitative trait loci (eQTL) analysis was used to determine whether *CaMK2A* SNPs that are associated with AD risk are differentially expressed in the brain among individuals with different genotypes.

## Materials and Methods

### Participants

Two independent samples of Chinese participants were recruited into this study. One cohort from eastern China was composed of 382 unrelated sporadic AD patients and 426 cognitively healthy individuals who were recruited from the Shanghai Mental Health Center and Tongde Hospital of Zhejiang Province. The cohort from southwestern China was enrolled at the Mental Health Center of Southwest China Hospital; this cohort was composed of 333 unrelated sporadic AD patients and 334 cognitively healthy controls. All patients were required to meet the following conditions: (1) diagnosed with AD according to the Diagnostic and Statistical Manual of Mental Disorders, Fourth Edition (DSM-IV) and the National Institute of Neurological and Communicative Disorders and Stroke/AD and Related Disorders Association (NINCDSADRDA; McKhann et al., 1984); (2) Han Chinese and over 65 years old; (3) defined as sporadic, i.e., their family history did not mention any first-degree relative with AD; and (4) Mini Mental State Examination (MMSE) scores for the following educational levels: illiterate <17, primary school education <20, middle school or above education <24. Patients with vascular dementia, Vitamin B12 deficiency, major depressive disorder, hypothyroidism or any other diseases that may cause cognitive decline were excluded. The healthy controls arose from the same base population and met the following criteria: (1) Han Chinese and over 65 years old; (2) confirmed as cognitively intact by MMSE screening; (3) no family history of AD; and (4) no neurodegenerative diseases, vascular diseases or systemic diseases. The patients and controls were independently diagnosed and screened by two psychiatrists who had worked in clinical practice for at least 5 years. Written informed consent was obtained from each participant prior to the performance of any procedure related to this study. In addition, the study protocol was approved by all the institutional review boards involved.

### Differential Expression Analysis of the CaMK2A Gene

To investigate whether *CaMK2A* was differentially expressed in the brain between AD patients and healthy controls, cross-platform normalized expression data for four brain regions [entorhinal cortex (EC), hippocampus (HP), temporal cortex (TC), and frontal cortex (FC)] were analyzed. Cross-platform normalization is a method that combines all expression data from multiple microarray studies into a unified dataset. Because cross-platform normalization removes artifacts between different platforms (batch effects) and preserves “real” biological differences between experimental conditions, this method is regarded to have a better performance in robust biomarker detection compared with the meta-analysis method (Taminau et al., 2014). Genes with a log 2 fold change greater than 0.1 (|log FC| > 0.1) and a false discovery rate (FDR) smaller than 0.05 (FDR < 0.05) were defined as being differentially expressed in AD patients. The above-mentioned analyses are accessible at the Alzdata.org web server^1^, and detailed information can be found in a previous study (Xu et al., 2018).

### SNP Selection and Genotyping

A total of 7 SNPs within *CaMK2A* (rs10051644, rs6869634, rs3797617, rs3756577, rs4958445, rs10515639 and rs6881743) were selected based on the following criteria: (1) SNPs are capable of tagging more SNPs based on the linkage disequilibrium (LD) pattern of the respective genes according to the data from the HapMap CHB dataset^2^ and 1,000 Genomes^3^, and (2) all eligible SNPs should have a minor allele frequency (MAF) >10% according to the HapMap CHB^2^ and dbSNP datasets^4^. Detailed information on each SNP is shown in Supplementary Table S1. Genomic DNA of all participants was extracted from peripheral blood using the AxyPrep™ Blood Genomic DNA Miniprep Kit (Axygen, USA). All SNPs were genotyped using the SNaPshot assay, as described in our previous studies (Wang et al., 2012; Bi et al., 2014).

### Brain eQTL (Expression Quantitative Trait Loci) Analysis for SNPs Associated With AD Risk

To further explore the differences in *CaMK2A* expression in various brain regions among individuals with different genotypes, we performed a eQTL analysis using the brain eQTL database^5^, which is a large exon-specific eQTL dataset covering 10 human brain regions [substantia nigra (SNIG), thalamus (THAL), inferior olivary nucleus (MEDU), putamen (PUTM), hippocampus (HIPP), temporal cortex (TCTX), intralobular white matter (WHMT), frontal cortex (FCTX), occipital cortex (OCTX), and cerebellar cortex (CRBL)]. More detailed information about this database can be found in the original literature (Ramasamy et al., 2014).

### Statistical Analysis

Hardy–Weinberg equilibrium (HWE) and allele and genotype frequency analyses were performed using SHEsis^6^. SNPs with a *P* value less than 0.001 were considered as a departure from the HWE (Wang et al., 2016b). In addition, we used Haploview 4.2 to perform a pairwise LD estimation and haplotype analysis. A strict FDR of 0.05 was applied as the multiple comparison correction to reduce the Type 1 error rate. All values were two-tailed, and we considered a *P-value* < 0.05 or the *P-value* threshold defined by FDR to be significant. All statistical analyses were conducted in the two independent cohorts separately, as well as in the combined Han Chinese population. Power calculations performed with Quanto 1.2.3 (Gauderman, 2002) were used to calculate the statistical power of the case-control sample size, assuming the prevalence of AD to be 5.56% in the Han Chinese population aged more than 65 years (Huang et al., 2019) and the OR to be 1.4. As the MAF of the selected SNPs fluctuated from 12% to 49%, the power of the sample ranged from 87.86% to 99.98%.

## Results

**Figure 1:**
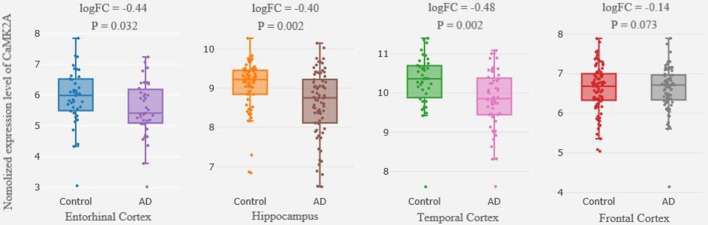
Differential expression of CaMK2A in brain between patients with AD and healthy controls. Each bar represents the average level of CaMK2A expression. Error bars represent the standard deviation of the mean value. Abbreviations: CaMK2A, calcium/calmodulin dependent protein kinase II alpha; AD, Alzheimer’s disease.

Differential expression analysis from the cross-platform normalized data showed that there were significant differences in *CaMK2A* expression in the HP, EC and TC between the AD patients and the control subjects (|log FC| > 0.1, *P* < 0.05); however, only the differences in the HP and TC remained following the multiple comparisons correction (FDR-corrected *P* = 0.034, *P* = 0.017, respectively). We failed to detect a difference in *CaMK2A* expression in the FC between the two groups (Figure 1).

**Figure 2:**
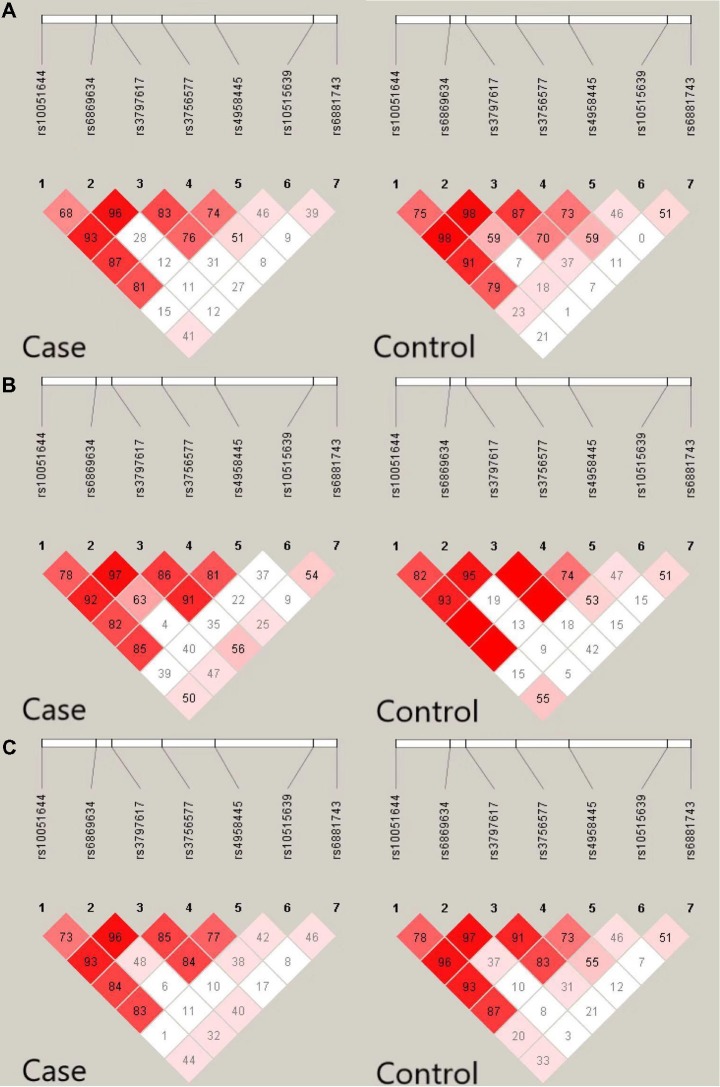
Linkage disequilibrium (LD) pattern of seven single nucleotide polymorphisms (SNPs) in the *CaMK2A* gene in AD patients and controls. Value in each square refers to *r*^2^ × 100. **(A)** LD pattern of seven SNPS in the *CaMK2A* gene in AD and controls from southwestern China. **(B)** LD pattern of seven SNPS in the *CaMK2A* gene in AD patients and controls from eastern China. **(C)** LD pattern of seven SNPS in the* CaMK2A* gene in AD and controls in the combined populations from eastern China and southwestern China.

In total, seven *CaMK2A* SNPs were analyzed in the current study, and the genotyping call rate of all individuals was 99.5%. No deviation from HWE was observed in any SNP analyzed. The genotype frequency of the rs4958445 polymorphism was shown to be significantly different between the AD and control subjects from southwestern China before (*P* = 0.013) and after the multiple comparisons correction (adjusted *P* = 0.034), whereas this difference was not detected in the cohort from eastern China (*P* = 0.403). When the two cohorts were combined, the results remained significant (*P* = 0.044) and were almost significant even after the multiple comparisons correction (adjusted* P* = 0.084). However, we failed to discover any positive association between other *CaMK2A* SNPs and AD in either the separate cohorts or the combined Chinese Han population. In addition, the allele frequencies of all the seven SNPs showed no significant difference between patients with AD and the control subjects (Table 1). The LD structures of the 7 SNPs were similar between the AD patients and controls in the separate cohorts and the combined cohort (Figure 2). Haplotype analysis indicated that the T-A-C-A-T-C-C haplotype and T-G-C-A-T-C-C haplotype in the southwestern cohort, consisting of rs10051644, rs6869634, rs3797617, rs3756577, rs4958445, rs10515639 and rs6881743, showed a significant association with AD (*P* = 0.037, *P* = 0.026, separately). In addition, our results also showed a significant association between the rs10051644T-rs6869634G-rs3797617C-rs3756577G-rs4958445C-rs10515639T-rs6881743C haplotype and AD in the eastern China sample (*P* = 0.045). However, no significant difference was detected for any haplotype in the combined Chinese Han population (see Table 2).

**Table 1:** Comparison of allele and genotype frequencies of the selected single nucleotide polymorphisms (SNPs) within CaMK2A between AD and healthy controls.

SNP ID	Populations	Allele	No. of samples		*P*-value	Genotype	No. of samples		*P*-value	Adjust *P*-value	HWE *P*-value^a^
			Patients	Controls			Patients	Controls			
rs10051644	Southwestern	C/T	79/583	70/596	0.411	CC/CT/TT	4/71/256	1/68/264	0.3712	0.597	0.151
	Eastern China	C/T	92/666	114/734	0.435	CC/CT/TT	5/82/292	6/102/316	0.7073	0.778	0.675
	Combined	C/T	171/1,249	184/1,330	0.927	CC/CT/TT	9/153/548	7/170/580	0.7606	0.864	0.176
rs6869634	Southwestern	A/G	154/506	151/517	0.752	AA/AG/GG	18/118/194	14/123/197	0.7398	0.95	0.434
	Eastern China	A/G	126/636	172/680	0.059	AA/AG/GG	6/114/261	13/146/267	0.1294	0.231	0.230
	Combined	A/G	280/1,142	323/1,197	0.295	AA/AG/GG	24/232/455	27/269/464	0.505	0.754	0.129
rs3797617	Southwestern	T/C	65/599	65/603	0.971	TT/CT/CC	3/59/270	1/63/270	0.5697	0.829	0.341
	Eastern China	T/C	72/690	89/763	0.505	TT/CT/CC	3/66/312	5/79/342	0.7657	0.856	0.796
	Combined	T/C	137/1,289	154/1,366	0.634	TT/CT/CC	6/125/582	6/142/612	0.845	0.948	0.689
rs3756577	Southwestern	A/G	188/476	179/487	0.558	AA/AG/GG	30/128/174	22/135/176	0.4899	0.695	0.676
	Eastern China	A/G	172/590	194/658	0.925	AA/AG/GG	22/128/231	28/138/260	0.8605	0.999	0.100
	Combined	A/G	360/1,066	373/1,145	0.673	AA/AG/GG	52/256/405	50/273/436	0.8646	0.793	0.433
rs4958445	Southwestern	T/C	254/410	231/437	0.164	TT/CT/CC	52/150/130	29/173/132	0.01676	0.034	0.011
	Eastern China	T/C	223/539	267/581	0.334	TT/CT/CC	37/149/195	41/185/198	0.4032	0.494	0.910
	Combined	T/C	477/949	498/1,018	0.729	TT/CT/CC	89/299/325	70/358/330	0.04424	0.084	0.058
rs10515639	Southwestern	T/C	160/504	176/492	0.344	TT/CT/CC	17/126/189	17/142/175	0.4753	0.833	0.092
	Eastern China	T/C	215/547	200/648	0.034	TT/CT/CC	30/155/196	25/150/249	0.102	0.163	0.687
	Combined	T/C	375/1,051	376/1,140	0.353	TT/CT/CC	47/281/385	42/292/424	0.6076	0.675	0.436
rs6881743	Southwestern	T/C	316/346	303/365	0.385	TT/CT/CC	78/160/93	72/159/103	0.6908	0.89	0.508
	Eastern China	T/C	328/434	369/483	0.915	TT/CT/CC	79/170/132	74/221/131	0.1151	0.233	0.278
	Combined	T/C	644/780	672/848	0.580	TT/CT/CC	157/330/225	146/380/234	0.2816	0.466	0.769

*AD, Alzheimer’s disease; CaMK2A, Calcium/calmodulin dependent protein kinase II. ^a^Hardy-Weinberg *P*-value was calculated in the controls group*.

**Table 2:** Results of the haplotype analysis between AD and control groups.

Populations	Haplotype	Frequency		*P*-value
		Patients	Controls	
Southwestern	CATGCCT	0.045	0.037	0.431
	TGCGCCC	0.190	0.211	0.378
	CATGCTT	0.094	0.015	0.381
	TGCGCCT	0.122	0.136	0.476
	CATGCCC	0.026	0.020	0.537
	TGCGCTT	0.085	0.082	0.846
	TGCATCT	0.090	0.086	0.811
	TGCATCC	0.099	0.064	0.026
	TGCACCC	0.020	0.023	0.644
	TGCGTCT	0.043	0.038	0.653
	TGCACCT	0.013	0.019	0.430
	TGCGTCC	0.026	0.027	0.953
	TACGTCT	0.045	0.026	0.072
	TACATCC	0.016	0.035	0.037
	TGCGCTC	0.085	0.100	0.360
	TACGTCC	0.014	0.017	0.599
	TACGCCC	0.016	0.017	0.977
	TACGCCT	0.026	0.019	0.448
	TGCATTC	0.030	0.029	0.922
Eastern China	CATGCCT	0.019	0.016	0.615
	TGCGCCC	0.263	0.267	0.883
	CATGCTT	0.019	0.018	0.867
	TGCGCCT	0.143	0.156	0.466
	CATGCCC	0.014	0.022	0.247
	TGCGCTT	0.081	0.064	0.212
	TGCATCT	0.068	0.071	0.796
	TGCATCC	0.067	0.073	0.796
	TGCACCC	0.020	0.028	0.347
	TGCGTCT	0.033	0.041	0.448
	TGCACCT	0.022	0.018	0.582
	TGCGTCC	0.024	0.022	0.721
	CATGTCT	0.009	0.014	0.334
	CGCGCCT	0.021	0.017	0.563
	TACGTCT	0.017	0.029	0.143
	TACATCC	0.022	0.021	0.873
	TGCGCTC	0.115	0.084	0.045
	TGCATTC	0.022	0.021	0.873
	CATGCTC	0.019	0.026	0.399
Combined	CATGCCT	0.031	0.024	0.250
	TGCGCCC	0.230	0.239	0.618
	CATGCTT	0.014	0.016	0.610
	TGCGCCT	0.136	0.148	0.338
	CATGCCC	0.021	0.026	0.425
	TGCGCTT	0.081	0.071	0.292
	TGCATCT	0.078	0.079	0.942
	TGCATCC	0.082	0.069	0.189
	TGCACCC	0.019	0.025	0.307
	TGCGTCT	0.036	0.040	0.637
	TGCACCT	0.019	0.019	0.931
	TGCGTCC	0.025	0.024	0.815
	CGCGCCT	0.015	0.013	0.626
	TACGTCT	0.027	0.027	0.951
	TACATCC	0.020	0.028	0.192
	TACGTCC	0.011	0.013	0.631
	TACGCCT	0.018	0.015	0.604
	TGCATTC	0.025	0.020	0.456
	CATGCTC	0.011	0.015	0.392
	TGCGCTC	0.100	0.091	0.417

*AD, Alzheimer’s disease*.

**Figure 3:**
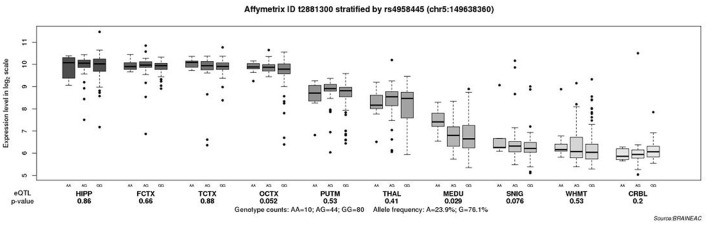
Association of rs5958445 with *CaMK2A* expression level in 10 brain regions (Affymetrix ID t2881300). Data were extracted from the BRAINEAC database (http://peana-od.inf.um.es:8080/UKBECv12/). Abbreviations: BRAINEAC, The Brain eQTL Almanac; CaMK2A, calcium/calmodulin dependent protein kinase II alpha; eQTL, expression quantitative trait locus; SNIG, substantia nigra; THAL, thalamus; MEDU, inferior olivary nucleus; PUTM, putamen; HIPP, hippocampus; TCTX, temporal cortex; WHMT, intralobular white matter; FCTX, frontal cortex; OCTX, occipital cortex; CRBL, cerebellar cortex.

To explore the role of various polymorphisms in the brain expression of *CaMK2A*, eQTL analysis was performed. Our results showed a significant association between the rs4958445 polymorphism and *CaMK2A* expression in the MEDU (inferior olivary nucleus, *P* = 0.029), and there was a clear trend toward significance in the OCTX (occipital cortex, *P* = 0.052). These results all emphasized that the expression of the AA genotype was higher than that of the A/G or GG genotype (Figure 3).

## Discussion

Ample evidence supports that genetic susceptibility may contribute to the development of AD (Gao et al., 2019). Numerous studies have indicated that the major pathological characteristics of AD include the abnormal gene expression of amyloid beta (Aβ) and tau protein in the brain (Hung et al., 2016; Li J. et al., 2017; Wang C. et al., 2018). Furthermore, it has been reported that *CaMK2A*, which has a potential link with Aβ production, has an aberrant expression in the brains of patients with AD (Ghosh and Giese, 2015). On this premise, we attempted to investigate the role of *CaMK2A* in AD. Our differential expression analysis confirmed that there are significant differences in *CaMK2A* expression in the brains of AD patients compared with control subjects, especially in the HP and TC. It has been well documented that the HP and TC are significantly associated with cognitive deficits, such as learning and memory (Ezzyat et al., 2018), and substantial research has also revealed abnormalities of the HP and TC in AD (Ye et al., 2019; Savioz et al., 2016). The finding that *CaMK2A* expression is altered in the HP and TC in AD implies that *CaMK2A* may be involved in the pathogenesis of AD.

To the best of our knowledge, this is the first study to investigate the association between* CaMK2A* and AD in the Chinese Han population. Herein, we genotyped seven *CaMK2A* SNPs in two independent Han Chinese cohorts from southwestern and eastern China. Our results showed that the genotype frequency of one *CaMK2A* SNP, rs4958445, is significantly associated with AD risk in populations from southwestern China and in the combined sample, and this association persisted in the southwestern cohort following the multiple comparisons correction. However, we did not detect any differences in the genotype frequency of SNPs within the eastern cohort. This disparity may be attributed to the different genetic backgrounds of the two cohorts (Wang J. et al., 2016).

To date, numerous preclinical studies have suggested a function of *CaMK2A* in spatial learning and memory (Matsuo et al., 2009; Moriguchi et al., 2014), which is a proposed functional endophenotype of AD (Nogueira et al., 2018). In addition, associations between several variants of *CaMK2A* and AD have been investigated in other ethnic populations. Bufill et al. (2015) screened seven SNPs of* CaMK2A* (rs2241695, rs3756577, rs3822606, rs13357922 and others) in the Spanish population and determined that the TT genotype at rs3756577 and the GG genotype at rs3822606 are underrepresented in Spanish AD patients. However, the link between rs3756577 and AD risk was not confirmed in our current study, and similar conflicting result has been reported by previous studies, indicating that the real functional variants may be associated with different SNPs in different populations (Wang et al., 2016b).

It has been widely accepted that haplotypes can be more specific risk markers compared with single alleles; therefore, the use of haplotypes can reduce false-positive associations in common psychiatric disorders (Zhang X. Y. et al., 2016). Since the seven markers analyzed in the present study were in the same haplotype block, we performed a seven-marker haplotype analysis, and our results showed that the T-A-C-A-T-C-C, T-G-C-A-T-C-C and T-A-C-G-T-C-T haplotype in the southwestern cohort and the T-G-C-G-C-T-C haplotype in the eastern cohort, which included rs10051644, rs6869634, rs3797617, rs3756577, rs4958445, rs10515639 and rs6881743, are significantly associated with AD risk. The fact that this association did not persist when the two cohorts were combined further suggests that people in different regions may have different genetic backgrounds. However, the results of the haplotype analysis in the independent sample all highlighted the relationship between *CaMK2A* and AD pathogenesis.

As is well known, numerous previous studies have suggested that there are structural and functional changes in certain brain regions in the case of AD, including the OCTX (Seidl et al., 2001; Kemppainen et al., 2006) and MEDU (Dowson, 1982). To detect the differential expression of SNP rs4958445 in the brain, eQTL analysis was performed. Our results indicate that the expression of rs4958445 in the MEDU and OCTX appears to be higher in individuals with the A/A genotype compared with those with the A/G or G/G genotype. In addition, the results of our present study indicate that the genotype frequency of SNP rs4958445 is significantly associated with the risk of AD in the Han Chinese population, and previous research revealed abnormalities in the MEDU and OCTX of AD patients. Taken together, our comprehensive analysis suggests that CaMK2A may regulate the structure and function of brain regions related to AD through altered gene expression, thereby leading to the pathogenesis of AD. However, the influence of *CaMK2A* on gene expression and its relationship with AD in the Han Chinese population should be verified in the future with a larger cohort.

The strength of the present study is that we combined our original data with previous relevant databases to perform a comprehensive analysis of the relationship between *CaMK2A* and AD for the first time. In addition, due to the regional differences in the Chinese Han population, we discussed the southwestern and eastern Chinese Han populations separately. However, several limitations of this study should be noted when interpreting the results. First, owing to the modest sample size, the conclusions that can be drawn from our data are limited. Second, only common variants of the *CaMK2A* were investigated; we did not analyze the rare variants of *CaMK2A*. Admittedly, this limitation precluded the detection of the active roles of such rare variants in the pathophysiology of AD. Third, although all patients and healthy controls were over 65 years old, we did not collect information about the age, sex and education level of the participants, which limited the information that was included in the association analyses. Therefore, future research involving larger samples of AD patients and focusing on a larger number of functional polymorphisms and their interactions, as well as other rare variants, may provide a more adequate understanding of the role of *CaMK2A* in AD. Such future studies may reveal the biomarkers of AD to facilitate early diagnosis and provide a more specific treatment of AD.

In summary, our case-control association study provides support that *CaMK2A* variants may play a major role in the pathophysiology of AD in the Chinese Han population. However, this finding remains preliminary due to the limited sample size and the fact that rare variants were not analyzed. Further independent validating studies and essential functional assays are warranted to confirm our observations and characterize the putative role of these genes in the pathophysiology of AD.

## Ethics Statement

This study was carried out in accordance with the recommendations of Ethics Committee of Shanghai Mental Health Center with written informed consent from all subjects. All subjects gave written informed consent in accordance with the Declaration of Helsinki. The protocol was approved by the Ethics Committee of Shanghai Mental Health Center.

## Author Contributions

XF, TL, D-FZ and CZ contributed to the overall design of the study. All authors got involved in the sample collection. XF and CZ undertook the statistical analysis, interpretation of data and wrote the manuscript. All authors contributed to the approval of the final manuscript.

## Conflict of Interest Statement

The authors declare that the research was conducted in the absence of any commercial or financial relationships that could be construed as a potential conflict of interest.

## Footnotes

^1^http://alzdata.org/Normalized_differential.html

^2^http://hapmap.ncbi.nlm.nih.gov/

^3^http://www.broadinstitute.org/mpg/snap/

^4^http://www.ncbi.nlm.nih.gov/SNP/

^5^http://peana-od.inf.um.es:8080/UKBECv12/

^6^http://analysis.bio-x.cn
