# Supplementary material for: A Comprehensive Analysis of the CaMK2A Gene and Susceptibility to Alzheimer’s Disease in the Han Chinese Population
Source: Front Aging Neurosci. 2019 Apr 11;11:84. doi: 10.3389/fnagi.2019.00084 (PMC6470288; doi:10.3389/fnagi.2019.00084)
Supplement: Supplementary file 1 [file Table_1.docx]

Table S1 Detailed information of 7 SNPs in CaMK2A gene.

| SNP rs no. | Allele | MAF^a^ | Chr. | Position | Annotation |
| --- | --- | --- | --- | --- | --- |
| rs10051644 | C/T | 0.17 | 5 | 150227185 | Intron 17 |
| rs6869634 | A/G | 0.24 | 5 | 150237156 | Intron 15 |
| rs3797617 | C/T | 0.12 | 5 | 150239936 | Intron 13 |
| rs3756577 | A/G | 0.16 | 5 | 150249081 | Intron 11 |
| rs4958445 | C/T | 0.27 | 5 | 150258797 | Intron 3 |
| rs10515639 | C/T | 0.24 | 5 | 150276863 | Intron 2 |
| rs6881743 | C/T | 0.49 | 5 | 150281027 | Intron 1 |

^a^Minor allele frequency (MAF) in CHB (Han Chinese in Beijing, China) was

obtained from HapMap (<http://hapmap.ncbi.nlm.nih.gov/>).

CaMK2A: calcium/calmodulin dependent protein kinase II alpha.
